# Retraction Note: Syndecan-1 suppresses cell growth and migration via blocking JAK1/STAT3 and Ras/Raf/MEK/ERK pathways in human colorectal carcinoma cells

**DOI:** 10.1186/s12885-024-12036-9

**Published:** 2024-03-01

**Authors:** Shaojun Wang, Xiaofei Zhang, Guimei Wang, Bin Cao, Hong Yang, Lipeng Jin, Mingjuan Cui, Yongjun Mao

**Affiliations:** 1 Department of Gastroenterology, The Affiliated Hospital of Qingdao University, 266000 Qingdao, China; 2 Department of Geriatrics, The Affiliated Hospital of Qingdao University, No.59 Haier Road, Laoshan District, 266000 Qingdao, Shandong China; 3 Emergency Department, The Affiliated Hospital of Qingdao University, 266000 Qingdao, China

**Retraction Note**: ***BMC Cancer*** **19, 1160 (2019)**

10.1186/s12885-019-6381-y

The Editors have retracted this article following an investigation by the Chinese Ministry of Science and Technology. The investigation found the data presented here is unreliable and that the corresponding author Yongjun Mao was not aware of the publication. The Editors therefore no longer have confidence in the integrity of the data in this article.

None of the authors have responded to any correspondence from the editor/publisher about this retraction.

